# Observations on the Use of the Plaster-Bandage in Ulcers

**Published:** 1803-02-01

**Authors:** 

**Affiliations:** Manchester


### *Observations on the Use of the Plaster-Bandage in Ulcers; communicated by* Mr. Simmons, *of Manchester.*

THE result of my early experience of the Plaster-Bandage, was published in the Annals of Medicine for the year 1797, under the title of "Observations on Mr. Baynton's Method of treating Ulcers on the Legs;" since that time, I have used it extensively, and with undiminished success. By these further opportunities, additional facts have been collected, all of which I shall now incorporate, and submit them, with deference, to the Public.

The Plaster-Bandage is adapted to almost every species of ulcer; but its utility is most conspicuous in that attended with callosity, in which, for the most part, it will accomplish a cure. But, in order to success, it will be proper,

1. To apply the strips spirally, yet so as to form a complete bandage.

2. To draw them sufficiently straight, to prevent the excess of the granulated above the general surface.

A due observance of the rules which are here laid down, would contribute gradually to supply the loss of tone in the limb; and also to hinder the excess of granulations, an obstacle which the formation of skin would otherwise have to encounter. And due care should likewise be taken not to form a complete circle, which would impede the circulation, and produce debility and œdema below.

The practice was restricted at first to inveterate ulcers upon the legs; but it has since been extended to recent ones, after the subsidence of the inflammation. And, in burns and scalds, it has been applied, both to promote the exfoliation of the slough, for which purpose it is better than emollient cataplasms; and also, to prevent or repress that exuberance of granulation, vulgarly denominated *proud flesh*, which escharotic dressings were formerly required to consume. Ulcers contaminated by a morbid poison will still demand their appropriate remedies; although, in these, the plasters will be preferred to cataplasms, emollient or digestive unguents, whereever their pressure can be borne. In the carious ulcer, they help to eject the decayed bone, and forward the disposition to heal.

The usual effect of the Plaster-Bandage, in the callous ulcer, is a mitigation of the pain, and an abatement of the inflammation; sometimes the effect is contrary, and both pain and inflammation are increased; but they are seldom of long continuance, and commonly yield in a few days to the repeated affusion of cold water. An objection of more weight is, the irregular ulceration brought upon the sound skin by the continuance of the pressure, which will need the direction of the plasters to be altered, or their use suspended, until the ulceration is healed and consolidated, by bathing it several times a day with the vegeto-mineral, or lime water, or daily dressing with the calamine cerate. The same remedies are also calculated to remove the pustular eruption, which is produced on the skin, in some constitutions, by the small proportion of resin contained in the plaster.

In scrofulous habits, the granulations are apt to grow loose and spongy, and to vegetate so luxuriantly, as to break through all resistance, and penetrate through the pores of the cloth; in that case, it will become expedient to touch them occasionally with the water of acetated litharge, or vitriol of copper, to limit their growth, and amend their disposition. But, before the cavity is filled up, the red precipitate will be better for destroying the surface of the ulcer, when the aspect of it is foul; or, by its becoming stationary, the cure hath ceased to advance. Those are all the accidents supervenient to the course, and their remedies, which experience hath taught. I shall next enumerate some considerable, though subordinate, advantages.

With regard to the frequency of dressing, every second or third day will be often enough, except where the discharge is profuse; because, a more frequent exposure would tend to disturb the formation of skin, or altogether destroy the beautiful film, which spreads from the circumference towards the centre of the ulcer, before its complete conversion into skin. Yet this saving of labour and of cost, is secondary to the economy and convenience of the patient becoming his own surgeon; as he may soon learn to apply the bandage himself, with accuracy; and also pursue his wonted occupation, without interrupting or retarding the cure.

As the operation of every bandage is mechanical, it is obvious that a preference will be due to that which is most certain in its agency. Upon this consideration, flannel is preferable to linen or callico, from being more elastic; and the Plaster-Bandage to all, from being still less liable to displacement. Nevertheless, the effects are not so palpable, as to preclude a diversity of sentiment concerning cerning them. My own I shall endeavour to illustrate by stating a case.

In that inveterate ulcer, which is situated anteriorly upon one of the legs, the ulcerated cavity is gradually obliterated by a double process; and the increasement of granulation is seconded conversely, by the absorption of the indurated and elevated integument. These opposite processes are continued, until the ulcerated and general surfaces are brought upon a level, and cicatrization only is wanted to a complete cure. Any bandage, bound on with care, would thus far answer the purposes intended; but afterwards, as no other could maintain the same evenness of surface, so none could give an equal facility to the expansion of skin. Accordingly, where the disposition to it is favourable, the skinning process now proceeds with great celerity, so great, that I have compared it to *crystallization* or *freezing;* though not from a supposition of identity, but to inculcate the necessity of preserving the surface of the ulcer in the condition just described. That this is the proper action of the bandage might be collaterally proved, by the uneven site of an ulcer, where several additional thicknesses of plaster will be wanted to fill up the vacuity, and render the compression equable. A method which may be pursued with advantage, and is most eligible in all those cases that need more pressure over a particular spot, than the Plaster-Bandage and exterior roller could safely exert.

Upon these grounds then I trust it will appear, that the healing of an ulcer is accelerated under this treatment, not by endeavouring to approximate the lips of it, which are immoveably fixed by chronic inflammation, but by hindering the exuberance of the granulations; or, if in excess, repressing them to a level with the general surface, and thereby facilitating the *crystallization* of skin.

This discovery will ever be esteemed of great value to the Public, inasmuch as sore legs are a very common and grievous complaint among the lower orders of society, and had, therefore, long constituted a large proportion of hospital practice. So burdensome were they become, that, for more than ten years, the rules of our Infirmary have excluded them its benefits as in-patients, after twice admission. This limitation did not proceed solely, however, from the intractable nature of such cases, but also from their furnishing a ready source of imposition to the idle and dissolute.

For the successful introduction of this valuable improvement, we are indebted to Mr. Baynton, of Bristol, whose

 claim claim to our gratitude is undiminished by any antecedent suggestion. The one to which I refer, is contained in the third volume of the Medical Transactions, in a paper on digitalis, by the late Dr. Darwin of Derby; which Mr. Baynton had not seen; and indeed it had not obtained notice, so that the practice would probably have been lost to the community, but for his descriptive account.

In the choice of materials for composing the plaster, it would appear, that any substance endowed with a quality moderately adhesive, would be equally eligible. Dr. Darwin recommended a composition of the minium plaster and carpenter's glue, for cheapness; and Mr. Baynton has used the adhesive plaster of the shops with a reduced proportion of resin. In some instances, where the skin is thin and delicate, even that small proportion of resin will excoriate, and oblige us to use the litharge plaster alone; which, when fresh and well prepared, especially during the summer months, will commonly prove sufficiently adhesive. I shall now conclude with a few more general observations.

Those Surgeons, who have been most attentive to the application of the bandage, have always been most successful in the treatment of ulcers. Wiseman well knew the principle of cicatrization, and exactly the degree of compression that might be safely and beneficially exerted; he says, "If the lips of the ulcer lie not level with the ulcer, it will not easily cicatrize; therefore you must make a more strict compression by bandage." Vol. i. p. 174. With the same intention, Mr. Else bound sheet-lead over the ulcer. But the Plaster-Bandage is preferable to every other, if applied in the manner directed by Mr. Baynton.

*December* 31, 1802.

